# Fibroblasts From Type 1 Diabetics Exhibit Enhanced Ca^2+^ Mobilization after TNF or Fat Exposure

**DOI:** 10.1371/journal.pone.0087068

**Published:** 2014-01-23

**Authors:** Nicholas R. Husni, Albert R. Jones IV, Amber L. Simmons, Barbara E. Corkey

**Affiliations:** Obesity Research Center, Evans Department of Medicine, Boston University School of Medicine, Boston, Massachusetts, United States of America; Dasman Diabetes Institute, Kuwait

## Abstract

The effects of cytokine and fatty acid treatment on signal transduction in dermal fibroblasts from type 1 diabetics and matched controls were compared. Chronic exposure to TNF, accentuated Ca^2+^ mobilization in response to bradykinin (BK) in cells from both controls and diabetics; responses were three-fold greater in cells from diabetics than in controls. Similarly, with chronic exposure to IL-1β, BK-induced Ca^2+^ mobilization was accentuated in cells from type 1 diabetics compared to the controls. Pretreatment with the protein synthesis inhibitor cycloheximide or the protein kinase C inhibitor calphostin C prior to the addition of TNF completely abrogated the TNF-induced increment in peak bradykinin response. Ca^2+^ transients induced by depleting endoplasmic reticulum (ER) Ca^2+^ with thapsigargin were also greater in TNF treated fibroblasts than in untreated cells, with greater increases in cells from diabetics. Exposing fibroblasts for 48 hours to 2 mM oleate also increased both the peak bradykinin response and the TNF-induced increment in peak response, which were significantly greater in diabetics than controls. These data indicate that cells from diabetic patients acquire elevated ER Ca^2+^ stores in response to both cytokines and free fatty acids,and thus exhibit greater sensitivity to environmental inflammatory stimuli and elevated lipids.

## Introduction

The inflammatory cytokines tumor necrosis factor-alpha (TNF-α) and interleukin-1β (IL-1β) are implicated in both type 1 and type 2 diabetes [1]. Elevated levels of these cytokines occur in newly diagnosed type 1 diabetics and in spontaneously diabetic mice [2]–[4]. Increased serum levels of TNF are also detected throughout the lives of both type 1 and type 2 diabetic patients [3] and in response to viral infection [5]. TNF and IL-1β impair glucose-stimulated insulin secretion [6], are directly toxic to pancreatic β-cells, and are implicated in autoimmune islet cell destruction [7], [8].

Despite abundant research, the etiology of type 1 diabetes remains unknown, however, there is support for a viral trigger for the disease [9]. Although debate continues concerning the nature of such a virus, abundant evidence links prior viremia and the onset of type 1 diabetes [10]. TNF is a pluripotent cytokine, producing effects on cells that range from the extremes of proliferation to apoptosis [11]–[14]. TNF initiates its cellular effects by binding to one of its two cell surface receptors: receptor p75 is thought to mediate the cytotoxic functions associated with TNF, while the p55 receptor mediates growth promoting and other cell stimulatory activities by several known intracellular signaling pathways including protein kinase C (PKC), phospholipase A_2_ (PLA_2_), mitogen activated protein kinase, and sphingomyelinase/ceramide [11]–[15]. IL-1β signaling has been shown to largely overlap the pathways used by TNF, and the two cytokines have many of the same effects on cells despite the fact that they bind to different plasma membrane receptors. Treatment of cells with TNF and IL-1β results in a strikingly similar pattern of phosphorylation and dephosphorylation, varying greatly from phosphorylation patterns obtained following treatment with another cytokine, epidermal growth factor [16].

Although type 1 diabetic patients may also have elevated serum levels of free fatty acids (FFA) or triglyceride, much less is known about how this may contribute to diabetic pathology than is known about the hyperglycemia-related pathologies. Even in non-ketotic states, type 1 diabetics have dyslipidemia, or elevated levels of FFA in serum [17]. Following insulin-induced hypoglycemia, stimulation of type 1 diabetics with epinephrine results in increases in FFA greater than in controls subjected to the same maneuver [18], [19]. Short term ketosis in type 1 diabetics is associated with almost doubled plasma FFA concentrations [20]. In addition to these few studies in type 1 diabetics, certain FFA have been shown to have effects on non-diabetic cells, ranging from modulation of intracellular Ca^2+^ homeostasis [21], [22] to activation of the nuclear transcription factor NF-κB and alteration of gene expression [23], [24]. Elevated plasma FFA, particularly saturated FFA, have been shown to induce islet inflammation [25]. Elevated extracellular FFA results in increased cytosolic long chain CoA, the effects of which may include modulating PKC activity, intracellular protein trafficking, G-protein activity, endoplasmic reticulum (ER) Ca^2+^-ATPase activity, expression of acetyl-CoA carboxylase, and peroxisome proliferation [24], [26], [27].

Inflammatory cytokines also affect lipid synthesis and metabolism. In rat liver, TNF increases hepatic fatty acid synthesis and lipid secretion [28]–[30]. Within 90 minutes, TNF treatment causes increases in hepatic citrate levels. The rise in citrate should elevate cytosolic long chain acyl CoA levels because citrate activates acetyl-CoA carboxylase which converts acetyl-CoA to malonyl-CoA, and since malonyl CoA is an inhibitor of carnitine palmitoyl transferase 1, the transporter that moves long chain acyl-CoA into the mitochondria for oxidation. Endotoxin inhibits oxidation of FFA in rats: it is presumed that this effect is mediated through TNF and IL-1β (endotoxin is a potent stimulator of TNF and IL-1β production by macrophages, which are known to mediate many endotoxin effects) [31]. TNF can also increase cytosolic FFA content directly, by activating phospholipase A_2_ [13], [32], [33].

Bradykinin (BK) is a vasodilator that plays a role in the inflammatory process, mediating acute responses to injury such as vasodilation, edema, and pain. Binding of BK to the G-protein coupled B2 receptor subtype leads to the activation of the phospholipase-C/inositol 1,4,5-trisphosphate (IP_3_) cascade and subsequent release of Ca^2+^ from internal stores [34]–[36]. The B2 receptor pathway also leads to an acute burst of prostaglandin E_2_ production in fibroblasts [37]. TNF and IL-1β have been shown to potentiate BK responsiveness in varied experimental systems [38], [39]. BK is an effective receptor-mediated agonist that we used in our studies to mobilize intracellular Ca^2+^.

The focus of this work was to compare the effects of inflammatory cytokines and fatty acids on BK-induced Ca^2+^ mobilization in fibroblasts from people with and without type 1 diabetes. The hypothesis examined was that type 1 diabetics have altered cytokine-mediated signaling compared to controls. Altered cytokine sensitivity in type 1 diabetics could clarify one role that inflammatory cytokines play in the pathogenesis of insulitis and induction of autoimmune β-cell destruction. We show here that TNF treatment of fibroblasts from diabetic subjects increased Ca^2+^ responses to BK about threefold above control values and that relatives of patients exhibited intermediate responses. Our data also show a significant differences between control and type 1 diabetic fibroblasts in Ca^2+^ signaling following FFA treatment.

## Research Design and Methods

### Ethics Statement

This research meets all applicable standards for the ethics of experimentation and research integrity. This research involved no active patient participation. The authors had no contact or interaction with the donors therefore no consent was required. Because human tissue samples were obtained through a third party vendor (The Coriell Institute for Medical Research, Camden, NJ), our research was exempt from the Boston University Institutional Review Board (H25457). The Coriell Institute ensured compliance with DHHS regulations for the protection of human subjects (45CFR Part 46). Human tissue was handled solely by the authors of this paper in our country of residence.

### Cell Cultures

Dermal fibroblasts were obtained from the Coriell Institute for Medical Research, Camden, NJ. Fibroblasts were obtained from 7 apparently normal donors, 10 donors identified as type 1 diabetics, and 3 non-diabetic siblings of the diabetic donors. Diabetic donors were matched to controls based on age, gender and race.

### Fibroblast Growth and Preparation

Cells were grown in Minimal Essential Medium (MEM) with Earle’s salts, 2X concentration of essential and non-essential amino acids for MEM, 1X MEM vitamin solution, 1X antibiotic/antimycotic, and 20% fetal bovine serum (FBS) from Hyclone Laboratories, Inc. (Logan, UT). All other cell culture solutions were purchased from Gibco Life Technologies (Gaithersburg, MD). Upon confluence, cells were removed from culture flasks by incubating for 1 minute in 0.7 mM EDTA in Dulbecco’s phosphate buffered saline (PBS; pH 7.4), and then for 2–5 minutes in 0.25% trypsin. Cells were then washed with PBS/EDTA and used for experiments, or passaged in a ratio appropriate to the culture’s growth rate.

### Cell Treatments

All experiments were performed in serum free media. Unless otherwise noted, the concentrations of TNF and IL-1β (both purchased from Genzyme Corporation, Cambridge, MA) were 10 ng/ml (0.6 nM) and 1 ng/ml (60 fM), respectively. The exposure to cytokine treatment was 24 hours (unless otherwise indicated) before cells were removed for Ca^2+^ measurement, which was performed in the absence of cytokines. Cells were preincubated for 30 minutes with 20 µM indomethacin (Sigma Chemical Company, St. Louis, MO) and 100 nM Calphostin C (Calbiochem-Novabiochem Inernational, La Jolla, CA) prior to the addition of TNF. Cycloheximide (3 µg/ml; Sigma) was preincubated for 3 hours before the addition of cytokines. Glucose (6 or 11 mM final) and oleic acid (2 mM; Sigma) were preincubated for 24 hours before cytokine addition. Oleic acid (free acid) was prepared as a stock solution of 0.1 M in NaOH (pH 9), and complexed to 2% fatty acid free bovine serum albumin (BSA; Sigma) at a final concentration of 2 mM.

As all fibroblast cultures were grown and maintained in a medium containing 20% FBS, and all TNF treatments were done in serum free medium, a preliminary experiment was performed in order to confirm that our TNF solution was not simply replacing a serum component in otherwise serum-starved cells. The addition of FBS did not cause a significant change in peak bradykinin response above those not treated with FBS.

### Ca^2+^ Measurement

Cytosolic free Ca^2+^ was determined from changes in the excitation signals of the fluorescent indicator fura-2 at 340 and 380 nm, measuring emission at 510 nm, using a Hitachi F-2000 fluorescence spectrophotometer, as described previously [40] (Hitachi High Technologies Corp., Tokyo, Japan). Following trypsinization and washing with PBS/EDTA, fibroblasts were loaded with 1 µM fura-2 acetoxymethyl (AM) ester (Molecular Probes, Eugene, OR) in MEM culture medium containing 0.5% BSA for 15 minutes. Approximately 250,000 cells were suspended in modified Krebs-HEPES buffer containing 120 mM NaCl, 5 mM KCl, 5 mM NaHCO_3_, 2 mM CaCl_2_, 1 mM MgCl_2_, 1 mM Na_2_HPO_4_, 10 mM glucose, 10 mM HEPES, 0.05% BSA, and 10 µM sulfinpyrazone (to block active extrusion of the fura), pH 7.4. The maximum Ca^2+^/fura-2 and minimum free fura-2 signals were determined after addition of Triton X-100 to equilibrate Ca^2+^ across the plasma membrane. The minimum Ca^2+^/fura-2 and maximum free fura-2 were determined after addition of EGTA (plus Tris buffer to maintain pH). The cytosolic free Ca^2+^ concentration was calculated by measuring the 2 fura signals as a percentage of the maxima where the K_d_ is 225 nM as described previously [40]. Ca^2+^ transients were measured in suspensions of confluent fibroblasts between passages 7 and 30; no consistent passage-dependent variation in the responsiveness of the cells was observed over this range.

### Permeabilized Cell System

After detaching with trypsin/EDTA as described, cells were resuspended in a buffer containing 100 mM KCl, 22 mM NaCl, 5 mM KHCO_3_, 20 mM HEPES, 1 mM MgCl_2_, 6 mM KH_2_PO_4_, 4 mM MgATP, 12 mM creatine plus creatine phosphate, 50 µg/ml creatine phosphokinase, and 1 µM fura-2 free acid [21]. Antimycin A (0.2 µg/ml) and oligomycin (2 µg/ml) were added to inhibit mitochondrial Ca^2+^ uptake. Saponin (60 µg/ml) was added when indicated to permeabilize the cells. The Ca^2+^ concentration in the buffer was measured by the fluorescence of free fura-2 (1 µM).

### Analysis of Data

To determine statistical differences between groups, analysis of variance (ANOVA) was used with Tukey’s post-hoc test for comparison of independent groups when appropriate (unless otherwise noted). Error bars on the figures represent the standard error of the mean.

## Results

### TNF and IL-1β Altered BK Responses in Fibroblasts from Control and Diabetic Donors

**Figure 1:**
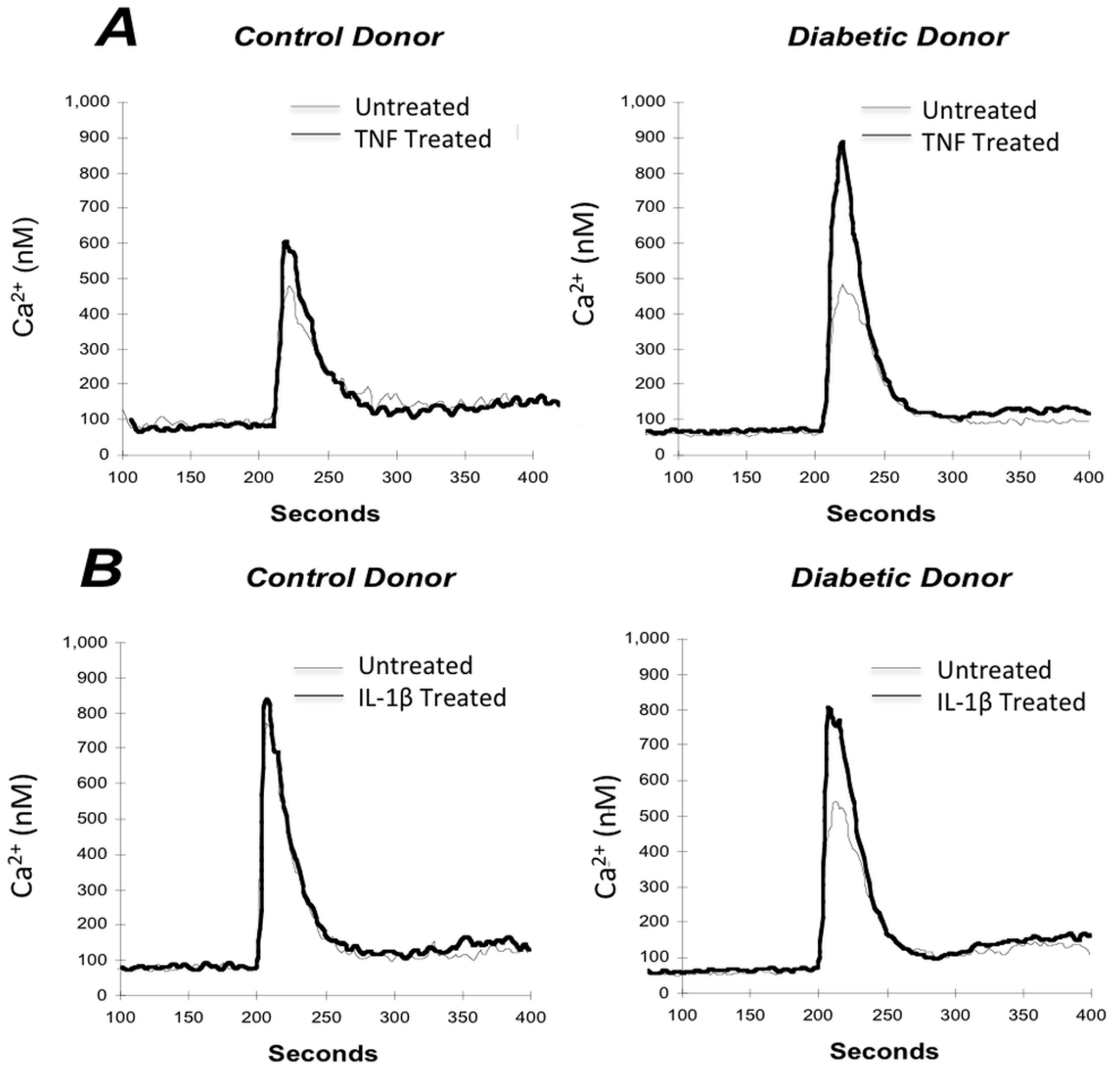
Representative traces of the effect of 24 hours of exposure to TNF (0.6 nM) or IL-1β (60 fM) on BK-induced Ca^2+^ mobilization. **A.** Fura loaded fibroblasts from control and type 1 diabetic donors, previously exposed to 0.6**B.** Fura loaded fibroblasts from one representative control and type 1 diabetic donor, previously exposed to 60 fM Il-1β for 24 hrs, were stimulated with 1 µM BK at 200 seconds. (Similar traces were obtained for each control, relative, and T1D sample and the summarized data is shown in Figs. 4 and 8).

TNF treatment had neither an acute effect on human fibroblasts on Ca^2+^ levels nor on BK-induced Ca^2+^ mobilization (data not shown). Figure 1A shows a representative trace that illustrates the the raw data obtained and the pattern of TNF (0.6 nM)-potentiated BK-induced Ca^2+^ mobilization (compare heavy line (treated) with fine line (untreated)). This figure shows representative traces from control and diabetic donors. In both control and diabetic donors, TNF treatment augmented BK-induced Ca^2+^ mobilization, although to a much greater extent in the diabetic donor (right panel). Similar sensitivity of diabetic donors were seen with 24 hours of IL-1β treatment (60 fM). Figure 1B shows representative traces in fibroblasts from control and diabetic donors in which IL-1β, like TNF, caused a greater increase in peak BK response in the diabetic donor whereas the response of the control was greater without treatment and not affected by IL-1β. These illustrations suggest that the enhanced cytokine sensitivity observed in diabetic fibroblast responses may not be limited to TNF but may reflect a general response to cytokines. However, further experiments were done with only TNF.

### Time and Concentration Dependence of TNF Treatment to Affect BK-induced Ca^2+^ Mobilization

**Figure 2:**
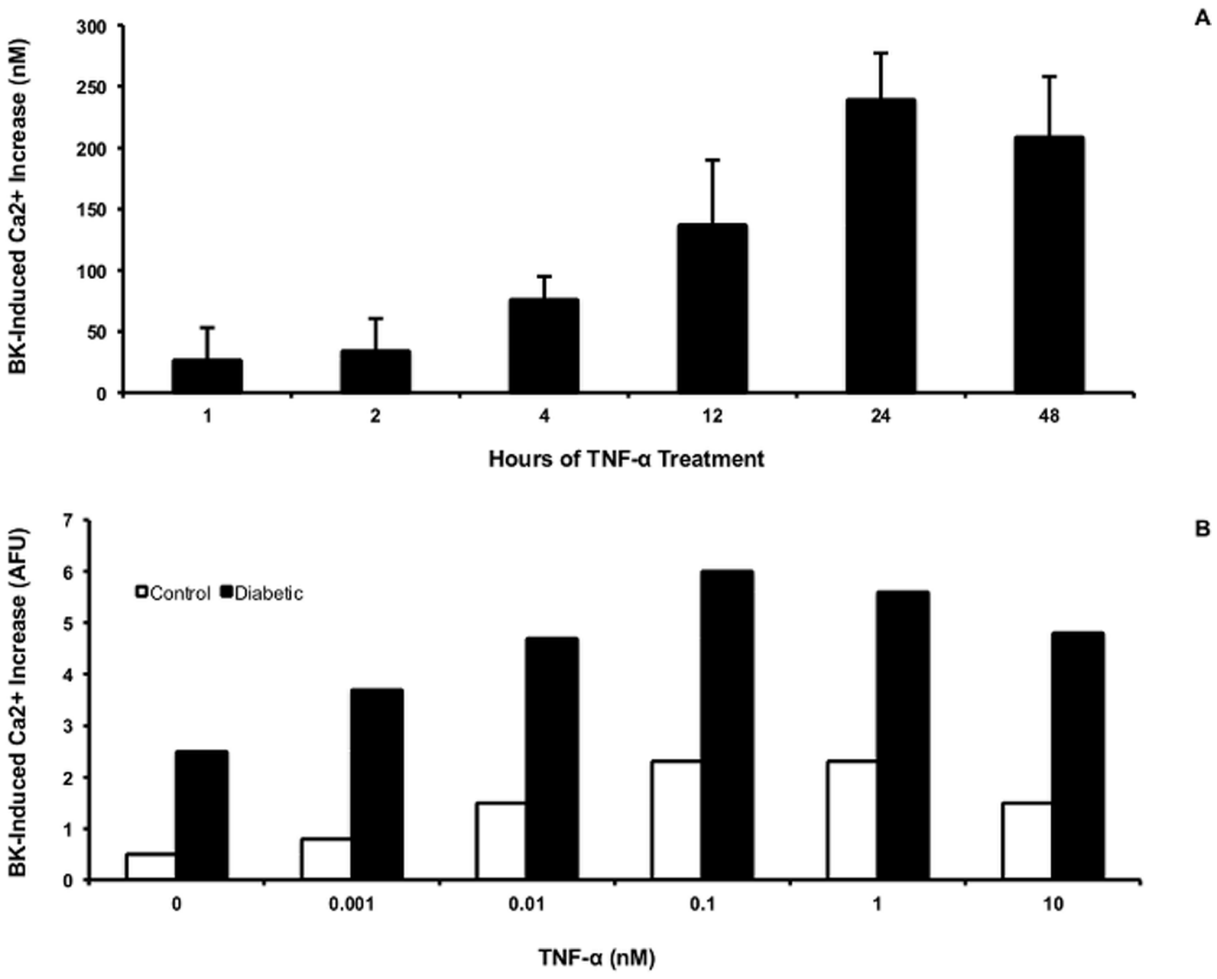
Time course (A) and concentration dependence (B) of effect of TNF pre-treatment on peak BK responses. A. Fibroblasts from 3 different donors were treated with TNF (0.6 nM) for 1 to 48 hours. Cells were loaded with fura as described in methods and tested with BK. Each bar represents the mean ± SEM of between 2 and 6 separate determinations. B. A single experiment performed in triplicate with fibroblasts from one control and one diabetic subject.

To determine the optimal time required for TNF to induce the peak BK response, time course series were performed in fibroblasts from 3 different donors, in which the cells were treated with 0.6 nM TNFfor 1, 2, 4, 12, 24, or 48 hr. Figure 2A shows the results of these experiments; each bar on the graph represents the mean of 2 to 6 separate determinations. In these donors, a TNF-induced increment in peak BK response could be seen within a few hours of treatment. A maximum increment was achieved by 24 hr that did not diminish significantly by 48 hr of treatment. All further incubations were for 24 hr.

The observation that time was required for TNF treatment to induce a change in peak BK response in fibroblasts (Figure 2A) suggested that the BK response might be dependent on the synthesis of new proteins. Cycloheximide, an inhibitor of protein synthesis, was employed to determine if synthesis was required for TNF to have its effect. There was no effect of TNF on fibroblasts pre-treated with cycloheximide indicating that expression of new proteins was needed for the observed effect (data not shown).

Evaluation of the concentration dependence of the stimulatory effect of TNF on the BK response (Figure 2B) indicated that the peak response occurred between 0.1 and 1 nM. Further experiments were performed at 0.6 nM TNF based on this experiment.

### Ca^2+^ Responses to BK in Control and Diabetic Fibroblasts: Effect of TNF Treatment

**Figure 3:**
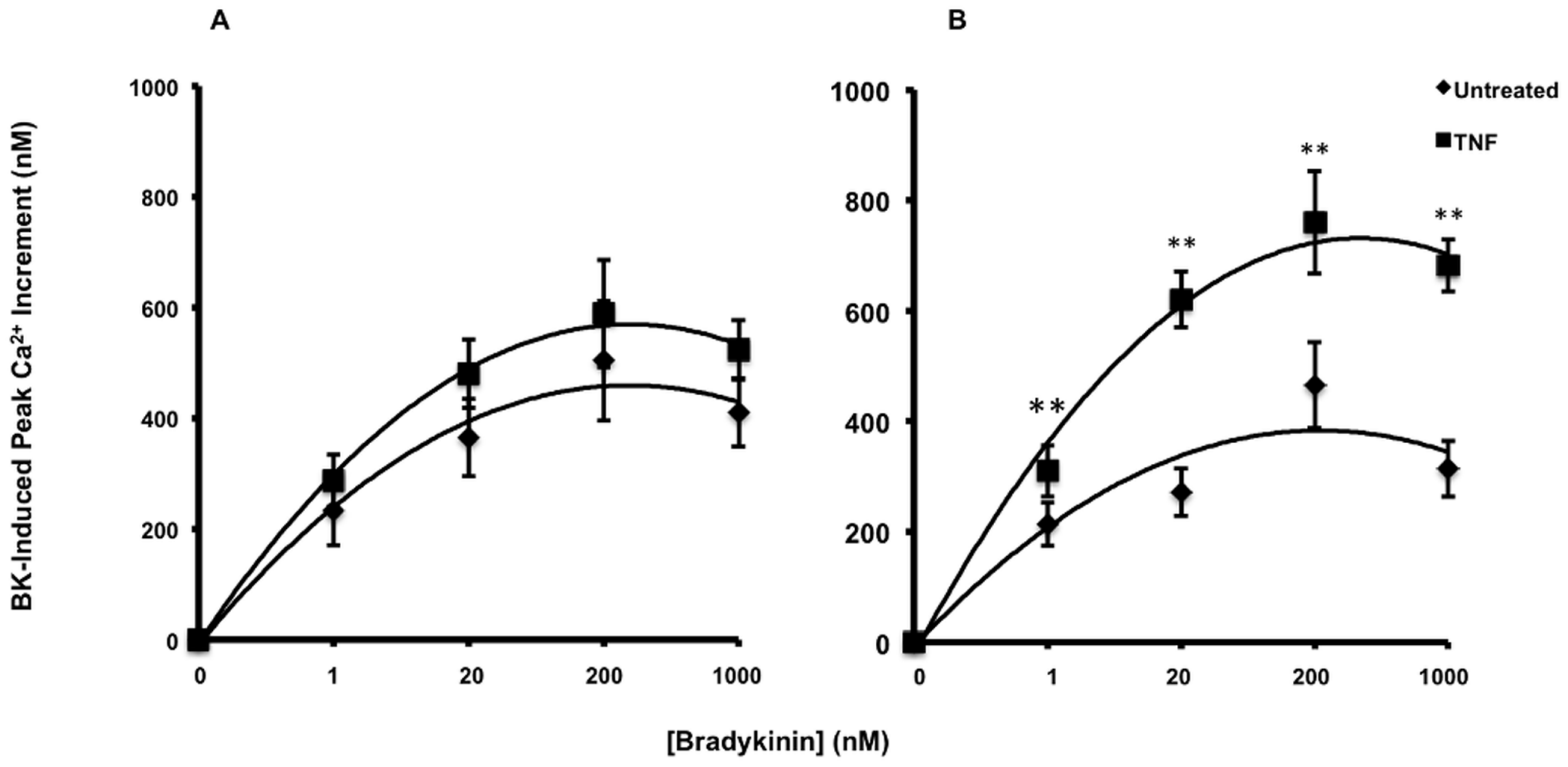
Summary of incremental effect of BK on Ca^2+^ mobilization in control (A) and T1D (B) fibroblasts. The change in peak response to BK before and after TNF treatment was measured in fibroblasts from 7 control (A) and 10 diabetic (B) donors (3–8 separate experiments per donor). Each point represents the mean ± SEM of 14 to 34 separate determinations. *Indicates that control is significantly different from diabetic (ANOVA *p*<0.005).

The concentration for maximal BK responses was tested between 0 and 10 µM and data are summarized in Figure 3. The concentration of BK where peak responses occurred were not altered by TNF in either control or diabetic cells. Subsequent experiments were performed with 1 µM BK.

A major difference between control and diabetic cells can be seen clearly from the average TNF-induced increment in peak BK response that exceeded 100 nM in all subjects (Figure 3A). However, cells from donors with diabetes showed a striking 3-fold greater effect of TNF than control cells (Figure 3A, black bars).

Because of the important signal transducing properties of the sustained phase of the Ca^2+^ response, the effects of TNF treatment on the increment in steady state, or final equilibrium, caused by BK were examined (Figure 3B). TNF pre-treatment also caused significant elevations in steady state Ca^2+^ during the sustained phase of the BK response in both control and diabetic donors which was significantly higher in the TNF pretreated diabetic fibroblasts than in the controls (*p*<0.005, Figure 3B).

### Comparison of BK Responses of Type 1 Diabetic, Non-diabetic Siblings of Diabetics and Controls

**Figure 4:**
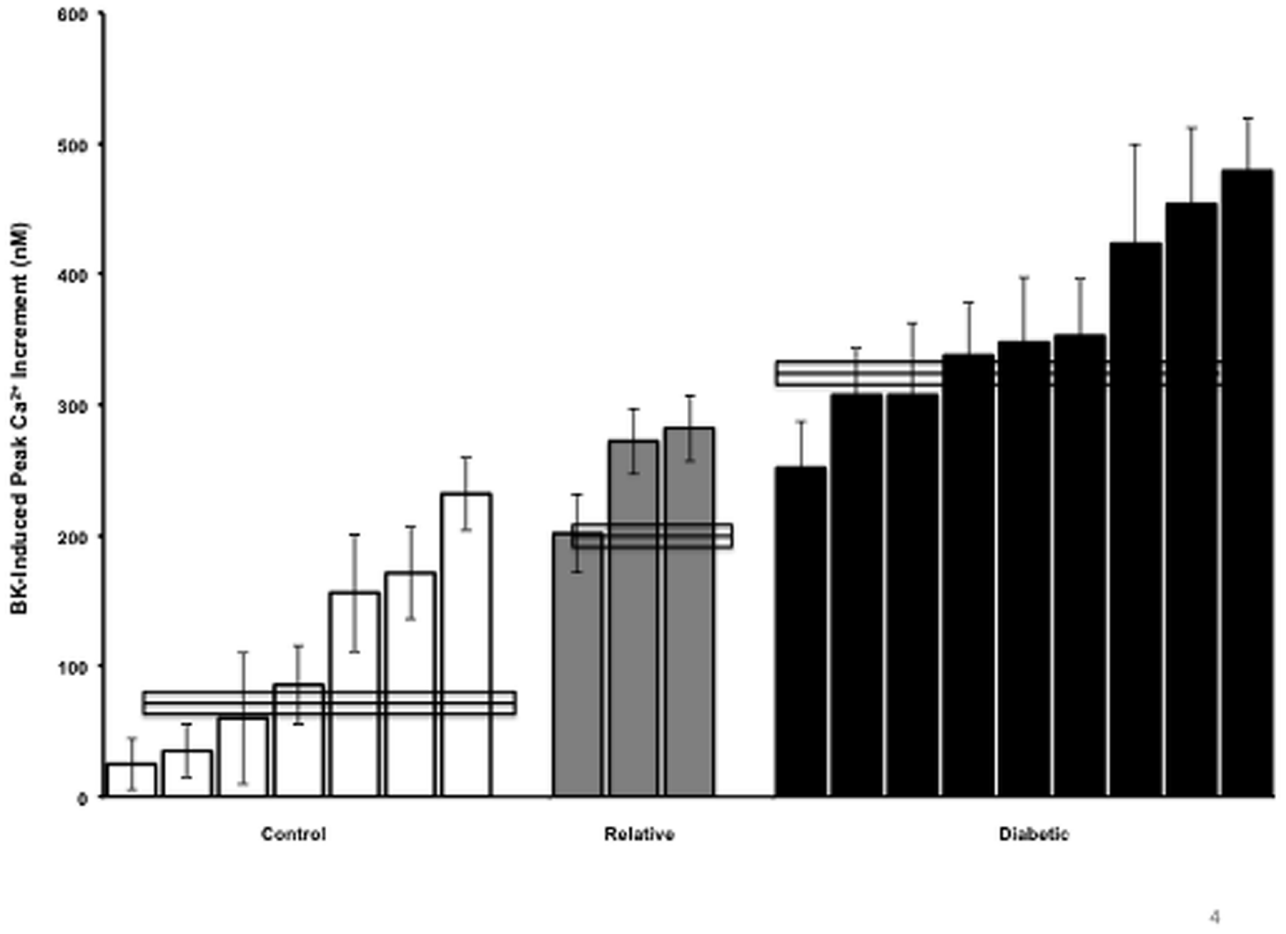
Comparison of TNF (0.6 nM for 24 hours) effects on BK-induced Ca^2+^ mobilization in type 1 diabetics, their siblings of type 1 diabetics, and control human fibroblasts. The increment in peak response to BK following TNF treatment was determined in 7 control donors, 3 non-diabetic siblings of type 1 diabetics, and 10 diabetic donors (3–8 separate experiments per donor). Each bar represents the mean of 6 to 18 separate determinations. The boxes across each donor group represent the mean ± SEM for each group of donors. Control, diabetic and sibling groups were significantly different from each other (ANOVA *p*<0.001).

The pathogenesis of type 1 diabetes indicates that there is a strong genetic component to the disease [41]. Cells from three non-diabetic siblings of the 10 previously described diabetic donors (all three from different families) were obtained to determine whether the fibroblasts from the non-diabetic siblings more closely resembled diabetics, or controls without a family history of diabetes. Figure 4 shows the TNF-induced increment in peak BK response in the 7 original control donors and the 10 original diabetic donors, plus 3 non-diabetic siblings of the diabetic donors. Interestingly, cells from donors whose siblings are diabetic exhibited a response that fell in between that of cells from donors with vs. without diabetes (*p*<0.001 between any group). This intermediate Ca^2+^ response to bradykinin from siblings of diabetics suggests that Ca^2+^ mobilization can be altered even in the presence of apparently healthy insulin response to glucose.

### Basal Ca^2+^ in Control and Diabetic Fibroblasts: Effect of TNF Treatment

The effect of TNF treatment on basal Ca^2+^ was determined in fibroblasts from 7 control and 10 diabetic donors (a mean of 96 to 105 separate determinations). In control donors, TNF did not significantly affect basal Ca^2+^ concentrations (71±2 nM pre- vs 74±2 nM post- 24 h TNF). In donors with diabetes, basal Ca^2+^ was lower inititially and was increased by TNF from 60±2 nM to 70±3 nM (*p*<0.001). Before treatment differences between fibroblasts from donors with and without diabetes were statistically signficiant (*p*<0.001).

### TNF Increased the Endoplasmic Reticulum (ER) Ca^2+^ Pool by Facilitating Influx

**Figure 5:**
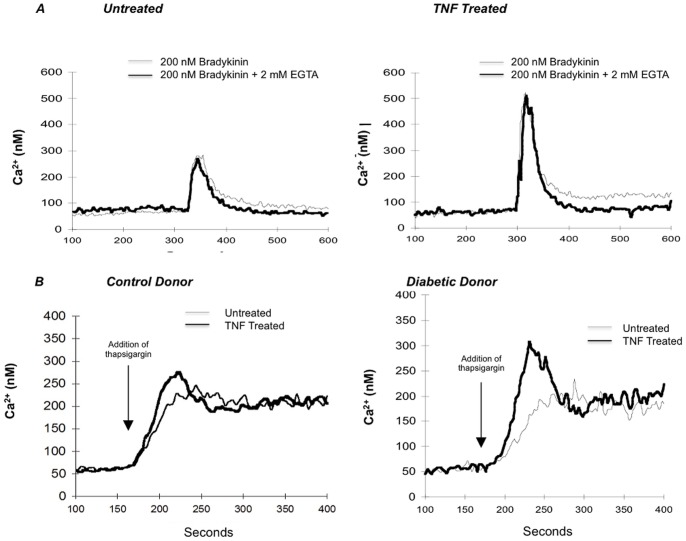
Representative traces to determine the source of TNF-induced increases in Ca^2+^ in response to BK. A. Untreated and TNF-treated (0.6 nM for 24 hours) fibroblasts were exposed to 200 nM BK. Addition of 2 mM EGTA 10 seconds prior to BK stimulation did not affect the magnitude of the peak in either untreated or TNF-treated cells, but did eliminate the increase in final Ca^2+^ equilibrium. These are representative traces from one diabetic donor. B. Fura loaded fibroblasts (untreated and TNF treated) from one control and one type 1 diabetic donor were treated with 30 nM thapsigargin at 170 seconds to release Ca^2+^ from the endoplasmic reticulum stores. These experiments were repeated three times with similar results.

To determine whether the TNF-induced increase in the peak BK response was due to mobilization of Ca^2+^ from intracellular stores or extracellular Ca^2+^ uptake, the Ca^2+^ chelator EGTA was added to the extracellular media immediately prior to stimulation with BK. The peak response to bradykinin was unaltered in the presence of EGTA chelation of extracellular Ca^2+^ in both untreated cells and TNF-treated cells (Figure 5A). This indicated that extracellular Ca^2+^ did not contribute to the bradykinin-induced peak Ca^2+^ response. The sustained elevation in steady state Ca^2+^ normally seen following bradykinin stimulation was abrogated both in untreated and TNF treated cells. Presumably the sustained phase was due to entry of extracellular Ca^2+^.

Thapsigargin, an irreversible inhibitor of the ER Ca^2+^-ATPase which depletes ER Ca^2+^ stores by inhibiting Ca^2+^ transport into the ER, was used to determine if the size of the ER Ca^2+^ pool influenced the TNF-induced increment in peak BK response. Addition of thapsigargin released Ca^2+^ from the ER, resulting in a rapid transient rise of cytosolic Ca^2+^ (Figure 5B). In fibroblasts from both control and diabetic donors, thapsigargin-induced Ca^2+^ peaks were greater following TNF treatment compared to cells that were not treated indicating that TNF treatment increased the ER Ca^2+^ stores (Figure 5B). As with bradykinin-induced Ca^2+^ mobilization (Figure 2A), the difference between the peak Ca^2+^ concentration between TNF-treated and untreated was much larger in the cells from patients with type 1 diabetes (394±28) compared to the controls (127±20 *p* = 0.001).

**Figure 6:**
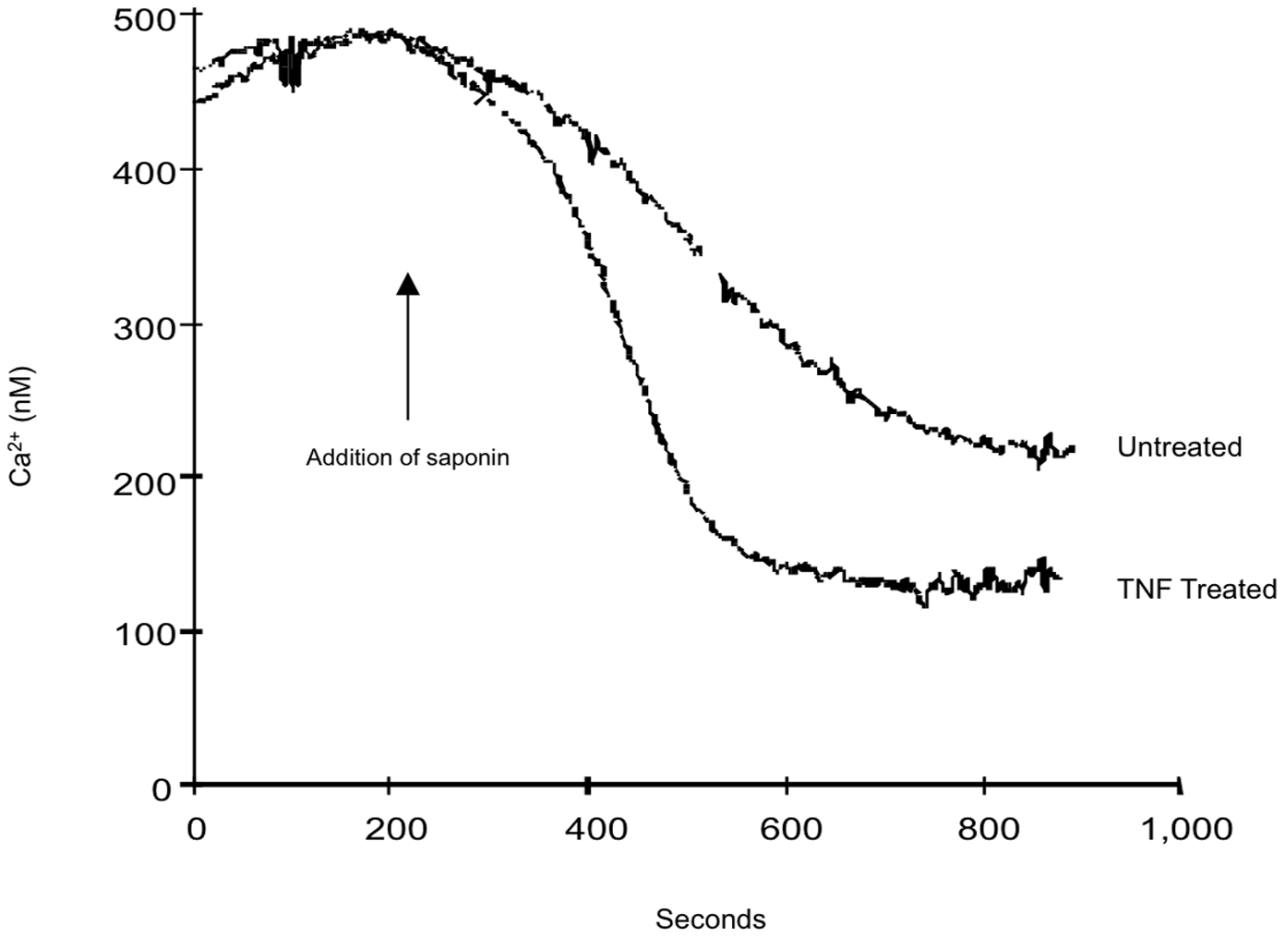
Representative trace illustrating the Ca^2+^ set point in permeabilized fibroblasts. Untreated and TNF-treated (0.6 nM for 24 hours) fibroblasts were suspended in an intracellular buffer containing 1 µM fura-2 free acid, and permeabilized with saponin (60 µg/ml) at 100 seconds. The cells took up Ca^2+^ from the buffer until steady state was reached. These experiments were repeated three times with similar results.

The ER Ca^2+^-ATPase regulates Ca^2+^ entry and, consequently, the size of the ER Ca^2+^ pools. To determine if the activity of the ER Ca^2+^-ATPase was affected by TNF treatment, fibroblasts were suspended in a buffer mimicking intracellular ion concentrations with no added Ca^2+^ and 1 µM fura-2 free acid (a fluorescent dye that fluoresces when it binds free Ca^2+^), and permeabilized with the detergent, saponin. The inhibitors oligomycin and antimycin A were added in order to inhibit the transport of Ca^2+^ into the mitochondria and limit the uptake of Ca^2+^ to the ER. As expected, the addition of saponin resulted in permeabilization of the cells and consequent rapid uptake of Ca^2+^ into the stores (Figure 6). The cells treated with TNF reached equilibrium faster, and the final equilibrium reached was lower. This suggested that TNF promoted Ca^2+^ transport into the ER via the Ca^2+^-ATPase. Figure 6 illustrates traces for paired, untreated and TNF-treated flasks of fibroblasts.

Changes in calreticulin binding have been shown to modulate the responses of Ca^2+^ mobilizing agonists [42], such that an increase in calreticulin levels could cause an increase in the size of the Ca^2+^ stores; however, western blot analysis showed no change in calreticulin levels after 24 hours of TNF treatment and there was no difference in calreticulin expression between control and diabetic fibroblasts (data not shown).

### Effects of Free Fatty Acids on Ca^2+^ Signaling

**Figure 7:**
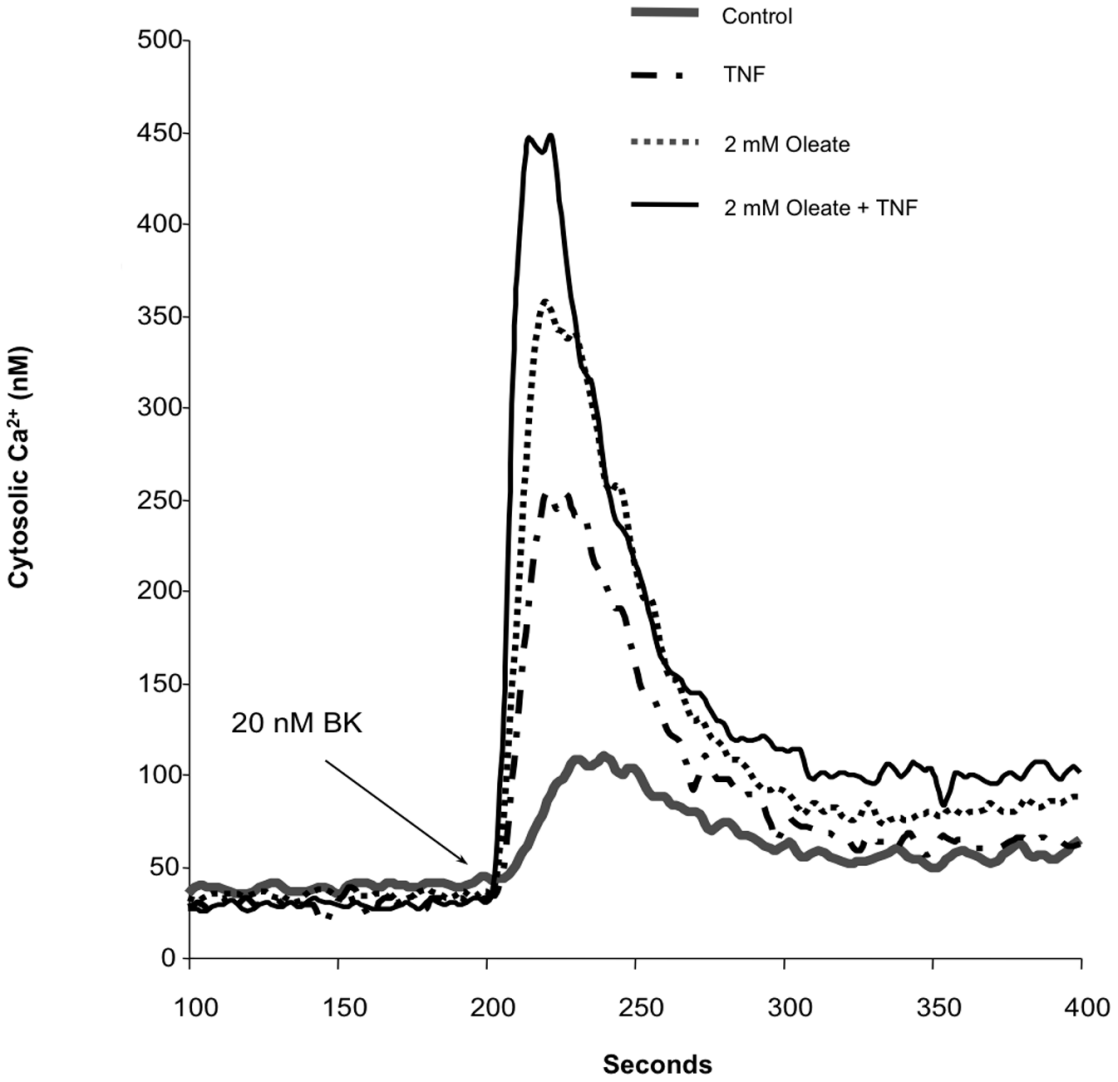
Representative traces of the effect of oleate and oleate plus TNF on peak BK response in fibroblasts from a single type 1 diabetic donor. These are representative traces of BK responses in fibroblasts from a single diabetic donor. Where indicated, 2 mM oleic acid was added 24 hours before, and throughout the subsequent 24 hour incubation. Cells in basal 5.6 mM glucose were treated with TNF, loaded with fura, and tested with BK as described in Figure 1.

The diabetic phenotype is associated with elevated blood glucose and lipid concentrations [43]. Circulating levels of FFA can impact cellular signaling and have the potential to affect Ca^2+^ mobilization. To examine the effects of FFA on BK-induced Ca^2+^ mobilization and on the TNF-induced increment, fibroblasts from 4 diabetic and 3 control donors were treated with 2 mM oleate for 48 hours. The fibroblasts from control donors were specifically selected from our collection of fibroblasts because they had responded to TNF treatment with only modest increases in peak BK response in previous experiments. TNF and oleate both augmented Ca^2+^ mobilization; simultaneous treatment of TNF and oleate led to the greatest effect. Figure 7 illustrates an experiment performed on fibroblasts from a single diabetic donor. As can be seen from the superimposed traces, each of the treatments had an effect on both peak BK response and the sustained plateau phase of the response following recovery from the peak. Traces from control donors showed little effect of TNF and a small effect of the diabetic medium.

**Figure 8:**
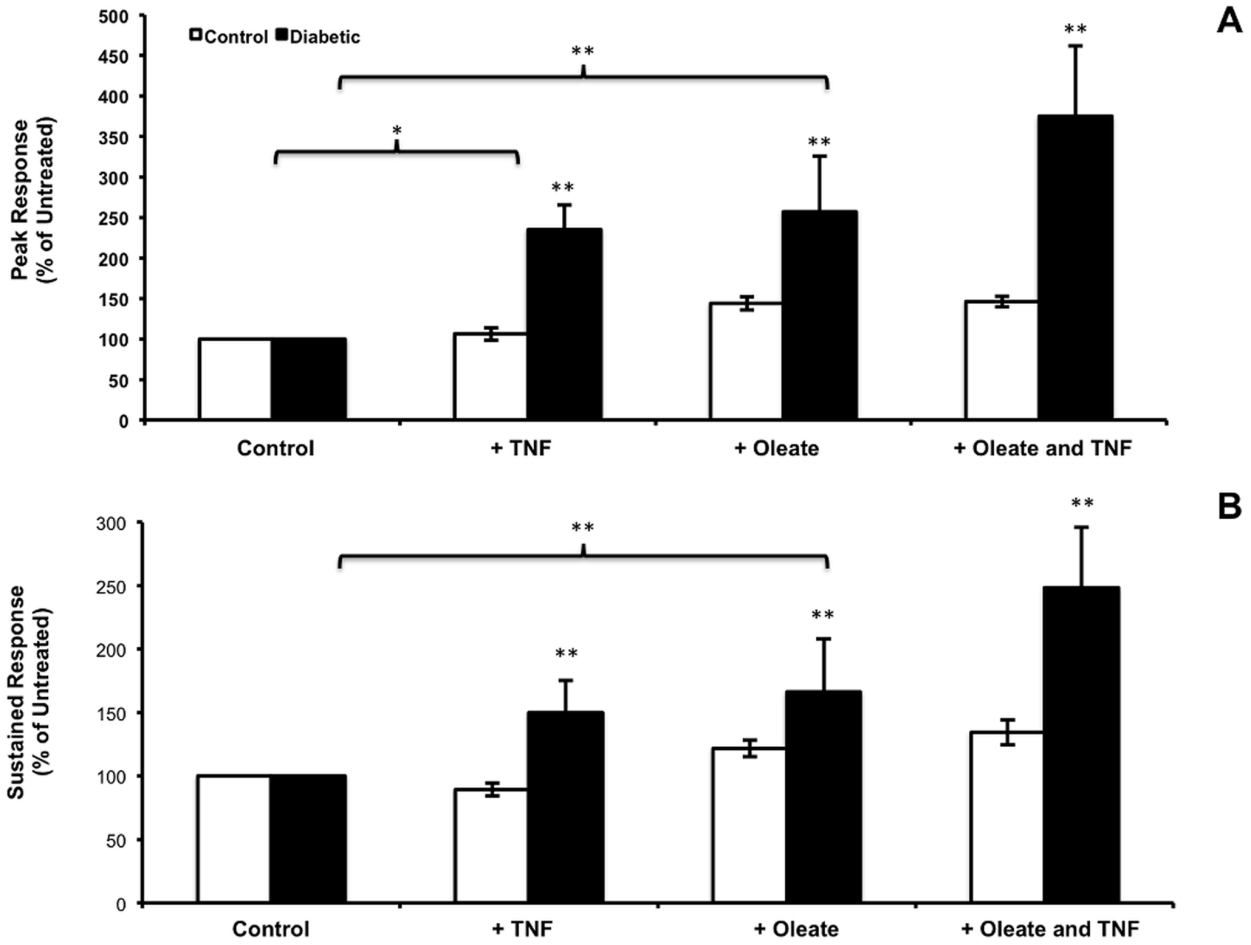
The effect of oleate and TNF on peak BK response in human fibroblasts. Fibroblasts from 3 relatively unresponsive controls and 4 diabetic donors were used. Where indicated, 2/or TNF was added 24 hours before, and throughout the subsequent 24 hour incubation. Cells were then loaded with fura and tested with BK as described in Figure 1. Data are expressed here as a percentage of the untreated condition (5.6 mM glucose). A. Both TNF and oleic acid had significant effects on peak BK response in diabetic cells (ANOVA *p*<0.05 and *p*<0.001 respectively). B. Oleic acid had a significant effect on the sustained steady state Ca^2+^ (*p*<0.001) in diabetic fibroblasts (*p*<0.001).

A summary of the results of experiments performed in 3 control and 4 diabetic donors is shown in Figure 8. Since there was some variation among donors, peak responses to BK were normalized to a percentage of that obtained with the control glucose alone. In addition, because FFA are added complexed to BSA it was necessary to add the same concentration of BSA to the control cells, however, the 2% BSA used in the media also binds a portion of the TNF. This may explain the failure of the control fibroblasts to respond to TNF treatment and the smaller response in the diabetic fibroblasts, in contrast to results obtained earlier (Figure 3).

The fatty acid-containing media had a significant effect on the sustained plateau in cytosolic Ca^2+^ following stimulation with BK (*p*<0.001), as shown in Figure 8B. Analysis of variance of the steady state data showed no significant effect of TNF in this system in either control or diabetic fibroblasts, probably due to BSA in the media.

## Discussion

Human skin fibroblasts from type 1 diabetic subjects exhibit several distinguishing features that differentiate them from control fibroblasts. These include an altered sensitivity to cytokines that results in greatly enhanced Ca^2+^ responses to BK and fatty acids. They also have a small but significantly lower basal Ca^2+^ that normalizes in response to cytokines. These features join several published studies documenting other differences between control and type 1 diabetic fibroblasts [44]–[46] and suggest possible additional markers of disease susceptibility that may be useful in applying preventive strategies to susceptible individuals [47].

**Figure 9:**
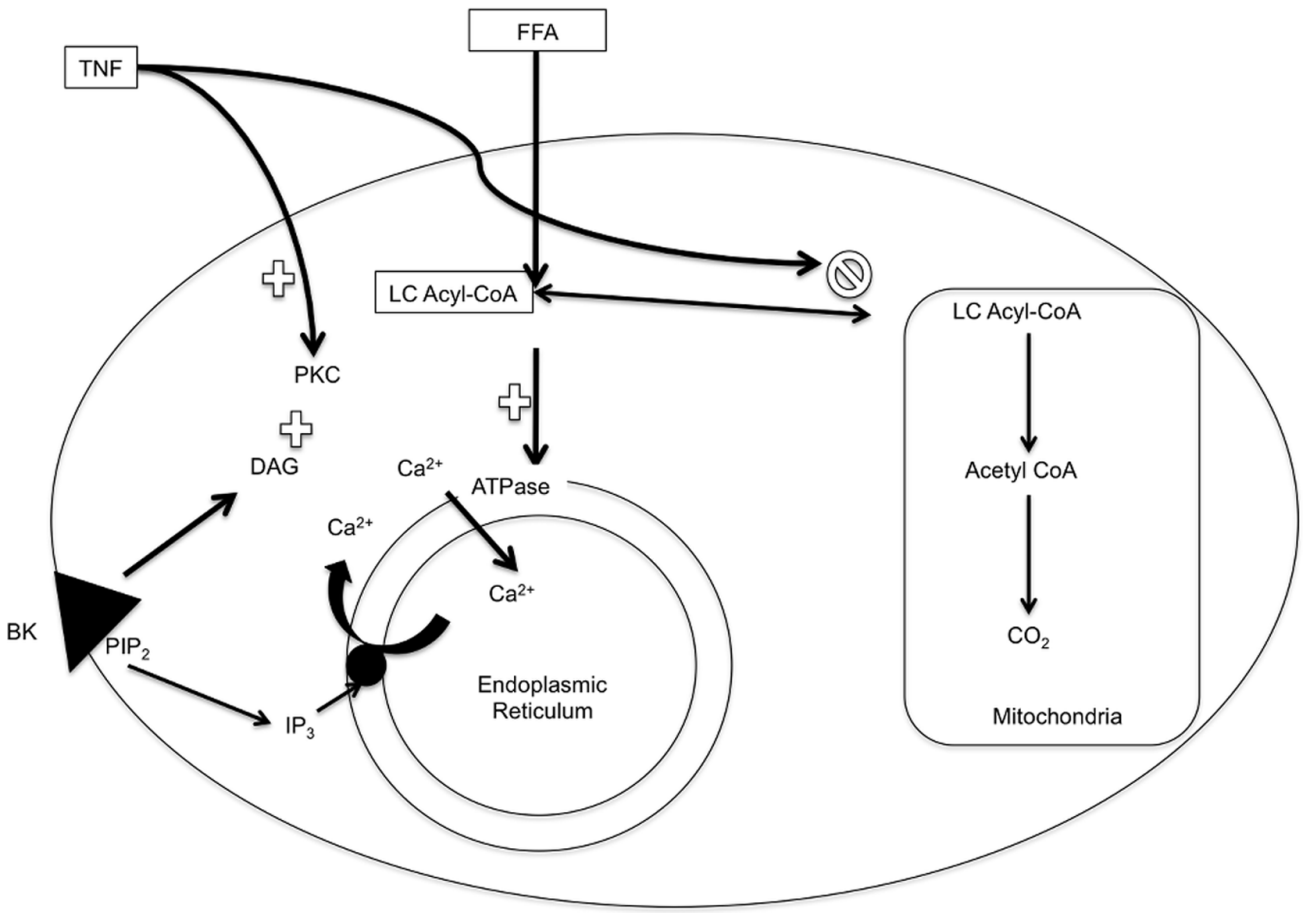
Model of TNF and oleate induced alteration in Ca^2+^ handling. TNF increases cytosol LC-CoA by inhibting its oxidation. Oleate increases LC-CoA via its cytosolic activation to oleoyl CoA. LC-CoA directly stimulates the ER Ca^2+^-ATPase to increase Ca^2+^ stores and activates PKC leading to an enhanced BK-induced signaling response.

The mechanism by which both TNF and a high fatty acid environment increase the peak Ca^2+^ response and steady state Ca^2+^ level following stimulation with BK is not known. These findings could have many separate causes, but also can be linked together in a simple speculative model (Figure 9) centered around cytosolic long chain (LC)-CoA. The model proposes that fibroblasts from type 1 diabetics are prone to greater elevation in cytosolic LC-CoA in response to cytokines or excess fatty acid. It has previously been shown that fibroblasts from people with diabetes incorporate more oleate into complex lipids than controls [46]. In addition, LC-CoA, the precursor for complex lipid formation, also directly stimulates Ca^2+^ uptake by the ER Ca^2+^-ATPase and increases Ca^2+^ stores [21]. Since TNF and IL-1β inhibit mitochondrial β-oxidation of free fatty acids [48], this would cause an increase in cytosolic LC-CoA and hence the size of the BK mobilizable Ca^2+^ stores. Fatty acids, by direct conversion to LC-CoA, also increase cytosolic long chain acyl CoA levels, increased Ca^2+^ stores, and enhanced BK response potentially by the same mechanism.

LC-CoA also has other modulatory roles in signal transduction including activation of PKC isoforms. PKC is known to play a role in TNF-induced signal transduction [13], [49], and we showed that calphostin C, which inhibits PKC by blocking the diacylglycerol binding site, effectively blocked the TNF-induced increment (data not shown). This suggested that PKC activity was necessary to observe a TNF-induced increment in BK response. In this model LC-CoA is the common signal that alters cytosolic Ca^2+^ stores via a direct effect on Ca^2+^-ATPase of the ER [21] and through activation of PKC. This could be linked to the reported abnormality in expression of FABP5 [44] that has been found in monocytes from type 1 diabetic subjects that may increase fatty acid availability in the cytosol for LC-CoA formation.

### Comparison of the Effect of TNF Treatment of Diabetics, Non-diabetic Siblings, and Control Donors

Analysis of the TNF-induced increment in peak BK response in all the donors surveyed (Fig 4) showed that they could be divided into 3 groups based on the level to which their fibroblasts responded to the TNF treatment. All but one of the control donors exhibited a TNF-induced increment between 200 nM and 300 nM calcium, while all but one of the diabetic donors exhibited an increment greater than 300 nM. Similarly, fibroblasts from only one control donor showed a Ca^2+^ increment greater than 200 nM. This artificial separation into three groups, according to the response of the cells to TNF treatment, suggests a genetic or epigenetic component. Studies currently underway will rederive the type 1 diabetic fibroblasts to determine if the characteristics are retained or lost following removal of the epigenetic changes [50]. Type 1 diabetics comprise a very small percentage of the general population, probably not greater than 1% [51], and 10 randomly selected type 1 diabetic fibroblast donors *all* exhibited greater effects of TNF than matched control donors. A trait present in 100% of such a small population may also occur with some frequency in the general population. If the cause of diabetes requires a combination of different factors, this can explain why two siblings who both carry a “diabetes gene” can be discordant for the disease. On the other hand, a person who does not carry the TNF-hypersensitivity trait may not become diabetic, whether or not exogenous stimuli such as a systemic viral infection occurs.

### Implications for Type 1 Diabetes

The data presented here indicate that fibroblasts from patients with type 1 diabetic display an altered response to BK in the presence of TNF and fatty acid. Together with data documenting elevated cytokines and free fatty acids in people with type 1 diabetes [2]–[4], [17], the results herein suggest that TNF and FFA may play a role in the etiology of many of the unique pathologies associated with diabetes ranging from autoimmunity to refractory wound healing. These two factors together, high cytokines and circulating fat, have an abnormal effect in cells from patients with type 1 diabetes who are much more sensitive than controls. Determination of this trait before development of diabetes could help to identify susceptible individuals prior to disease onset. Strategies to diminish this hypersensitivity or exaggerated Ca^2+^ signal transduction could lead to improved outcomes.
